# Associations between ambient air pollution and daily incidence of pediatric hand, foot and mouth disease in Ningbo, 2014–2016: a distributed lag nonlinear model

**DOI:** 10.1017/S0950268820000321

**Published:** 2020-03-04

**Authors:** Shaohua Gu, Decheng Li, Beibei Lu, Ruixue Huang, Guozhang Xu

**Affiliations:** 1 Ningbo Municipal Center for Disease Control and Prevention, Ningbo 315010, China; 2 Department of Occupational and Environmental Health, Xiangya School of Public Health, Central South University, Changsha 410078, China

**Keywords:** Air pollutants, HFMD

## Abstract

Hand, foot and mouth disease (HFMD) has high prevalence around the world, with serious consequences for children. Due to the long survival period of HFMD virus in ambient air, air pollutants may play a critical role in HFMD epidemics. We collected data on daily cases of HFMD among children aged 0–14 years in Ningbo City between 2014 and 2016. Distributed lag nonlinear models were used to assess the effects of particulate matter (PM_2.5_), sulphur dioxide (SO_2_), nitrogen dioxide (NO_2_) and ozone (O_3_) on the daily incidence of HFMD among children, with analyses stratified by gender and age. Compared with moderate levels of air pollution, high SO_2_ levels had a relative risk (RR) of 2.32 (95% CI 1.42–3.79) and high NO_2_ levels had a RR of 2.01 (95% CI 1.22–3.31). The RR of O_3_ was 2.12 (95% CI 1.47–3.05) and that of PM_2.5_ was 0.77 (95% CI 0.64–0.92) at moderate levels of air pollution. Specifically, high levels of SO_2_ and NO_2_ had RRs of 2.39 (95% CI 1.44–3.96) and 2.02 (95% CI 1.21–3.39), respectively, among 0–4-year-old children, while high O_3_ had an RR of 2.31 (95% CI 1.09–4.89) among 5–14-year-old children. Our findings suggest significant associations of high SO_2_ and NO_2_ levels and moderate O_3_ levels in HFMD epidemics, and also indicate that air pollution causes lagged effects on HFMD epidemics. Our study provides practical and useful data for targeted prevention and control of HMFD based on environmental evidence.

## Introduction

Hand, foot and mouth disease (HFMD) is a common illness caused by a variety of enteroviruses, such as enterovirus 71 or Coxsackie, and usually affects children [1]. The first outbreak of HFMD was reported in Canada in 1957 by Robinson *et al*. [2]. After that, a series of reports on individual cases of HFMD and larger outbreaks have been published across the world. In the United States, 63 cases were identified between November 2011 and February 2012, according to the U.S. Centers for Disease Control and Prevention (https://www.cdc.gov/). Asia was reported to have the most outbreaks of HFMD across the world. In China, a national survey from 2008 to 2012 estimated that ~15 million cases had been reported, with an incidence of 1.2 per 1000 person-years, and the disease is responsible for 350–900 reported deaths annually, predominantly among young children [3]. HFMD has also occurred in Japan during the past decade. Between April and September 2012, 2900 cases have been reported in children under 15 years of age [4]. Other Asian countries, including India, Singapore and South Korea, have also reported cases of HFMD [5–7]. HFMD is transmitted by faecal–oral, oral–oral and respiratory droplet contact [8]. Outbreaks can occur in the spring to fall and most cases occur in patients younger than 10 years [8]. Experimental studies have indicated that although this disease is self-limiting and the clinical symptoms are mild, such as rashes or mucosal herpes, severe complications, such as meningitis or encephalitis, occasionally occur. These can result in death, particularly among young children under 5 years of age [9]. The incubation period of HFMD is typically about 3 to 10 days [8, 10, 11]. To date, no effective vaccines have been developed against HFMD-related viruses. Hence, determining the risk factors so that we can establish an early warning system remains a crucial part of the measures to prevent and control HFMD outbreaks, and thus reduce the burden of the disease for children [12].

Air pollution has been reported to be a leading cause of global mortality. The World Health Organization estimates that ~7 million premature deaths were partly caused by air pollution in 2012. This corresponds to almost one in eight deaths globally (http://www.who.int/phe/health_topics/outdoorair/databases/FINAL_HAP_AAP_BoD_24March2014.pdf?ua=1). Epidemiological and clinical studies have demonstrated that exposure to air pollution, and particulate matter (PM_10_ and PM_2.5_) in particular, is associated with childhood diseases such as allergy, asthma and congenital hypothyroidism and increases the risks once a disease has been contracted [13–15]. These findings suggest that essential air pollution sources, such as nitrogen dioxide (NO_2_), ozone (O_3_) and sulphur dioxide (SO_2_) could also contribute to HDMF outbreaks. Susan *et al*. estimated that 9–23 million asthma cases may be attributable to air pollution, specifically O_3_ and PM_2.5_ [16]. Guoqi *et al*. analysed the effect of air pollution on HFMD, and found that low O_3_ levels increased the risk, whereas low PM_2.5_ levels protected against HFMD [17]. However, the results of our previous study did not suggest any relationship between exposure to PM_10_ and the incidence of HFMD [18]. The conclusions drawn from these data are inconsistent with respect to air pollution and the incidence of HFMD. Nonetheless, to date, only limited studies have been carried out on the relationship between air pollution and HFMD incidence. For example, SO_2_ has not yet been included in assessments of pollutants associated with HFMD, but it is considered a significant pollutant in both cities and rural areas [19]. We hypothesised that the incidence of HFMD may be attributable to exposure to four essential air pollutants (SO_2_, NO_2_, O_3_ and PM_2.5_). We used a distributed lag nonlinear model (DLNM) to analyse the associations between air pollution and the incidence of HFMD in Ningbo City between 2014 and 2016. This is the first analysis of the relationship between the incidence of HFMD and combinations of multiple air pollutants. We expect that the results of our study will be helpful for clarifying potential relevant factors and providing a reference for establishing an early warning system to prevent children from contracting HFMD.

## Methods

### Data collection

Ningbo City is a well-known economic centre and tourist destination in eastern China. Ningbo has an area of 9816 km^2^ and a population of 7.80 million (in 2016). The city is located near the coast, with an average annual temperature of 22 °C and an average relative humidity of 70%.

We obtained information regarding HFMD cases among children aged 0–14 years between January 2014 and December 2016 from the National Notifiable Disease Reporting System of the Ningbo Municipal Centre for Disease Control and Prevention. The standards for the diagnosis of HFMD were based on recommendations from the Chinese Ministry of Health [20]. We tried to ensure that our analysis was based on a stable population by only including the records of patients living in five central urban districts in our data set. To identify vulnerable populations, we reclassified the data by sex (male, female) and age (0–4, 5–14 years old).

We collected data on SO_2_, nitrogen dioxide (NO_2_), daily maximum 8 h average ambient O_3_ concentration (O_3_-8 h) and ambient fine particulate matter (⩽2.5 µm in aerodynamic diameter, PM_2.5_) measured at eight monitoring stations in the central urban area. These data were provided by the Ningbo Environment Monitoring Center. We averaged daily measurements from the eight monitoring stations to create a daily estimated exposure. The method has been reported previously [21, 22]. The daily meteorological data used in this study were published by the Ningbo Meteorological Bureau. They included daily average temperature, relative humidity and sunshine duration. These data were measured at the Yinzhou station, which is the only national weather monitoring station in the central urban area of Ningbo city.

### Data analysis

We used a DLNM to ensure that our model had sufficient flexibility to describe the lag time dimension of the exposure–response relationship. These are usually applied to the analysis of the relationship between air quality and disease incidence due to their ability to capture predictors at both the usual pace and with the additional dimension of temporal lag [23]. For example, this model has been applied previously to assess the effect of heat waves on cardiovascular daily mortality and temperature changes on the numbers of HFMD-related emergency room visits and morbidity [24, 25]. It has been suggested that this model can be used effectively to investigate associations between HFMD incidence and air pollution.

The model is defined by the following formula:
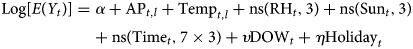
where *E*(*Y*_*t*_) is the expected number of HFMD cases on calendar day *t* (1,2,…1096) and *α* is the model intercept; we controlled for long-term and seasonality trends using a natural cubic spline (ns) with 7 degrees of freedom (df) per year for time (1,2,…1096), and adjusted for other environmental confounding variables using a natural cubic spline with 3 df for relative humidity (RH_*t*_) and sunshine duration (Sun_*t*_) [26, 27]. Although there is no consensus on how many knots are optimal, 7 per year has been justified as a balance between providing adequate control for seasonality and other confounding by trends in time, while leaving sufficient information from which to estimate exposure effects [27]. We controlled for the potential effects of temperature using a cross-basis function (Temp_*t,l*_), natural cubic splines with 4 df for the exposure–response relationship and natural cubic splines with 4 df for the lag–response relationship. We included dummy variables for day of week (DOW_*t*_) and holidays (Holiday_*t*_), which included vacation days for elementary schools and national public holidays. AP_*t,l*_ represented the bi-dimensional natural cubic spline of the exposure–response association between air pollutants and daily HFMD cases, and we used a non-linear model to determine the effects of air pollutants. We used a maximum lag of 14 days to capture any lagged effects of air pollution, considering that HFMD has an incubation period that is typically less than 14 days. The Akaike's information criterion for the quasi-Poisson model (Q-AIC) was applied to verify the optimal df of the models [28]. To account for autocorrelations in the residuals, we included autoregressive terms of the HFMD daily counts at lag 1 and 2 into the model based on an autocorrelation plot of the residuals.

To assess the effects of each air pollutant, we used the 1st percentile of each air pollutant as a reference value, and the effects of moderate and high levels of air pollutants were estimated by calculating the risk of HFMD cases at the 50th and 99th percentiles of each air pollutant relative to the reference value, with lags of 0–14 days. We performed all statistical analyses using the ‘dlnm' package in the R programming language (ver. 3.1.1; R Development Core Team, Vienna, Austria) and defined statistical significance as a two-tailed *P* < 0.05.

## Results

**Fig. 1.**
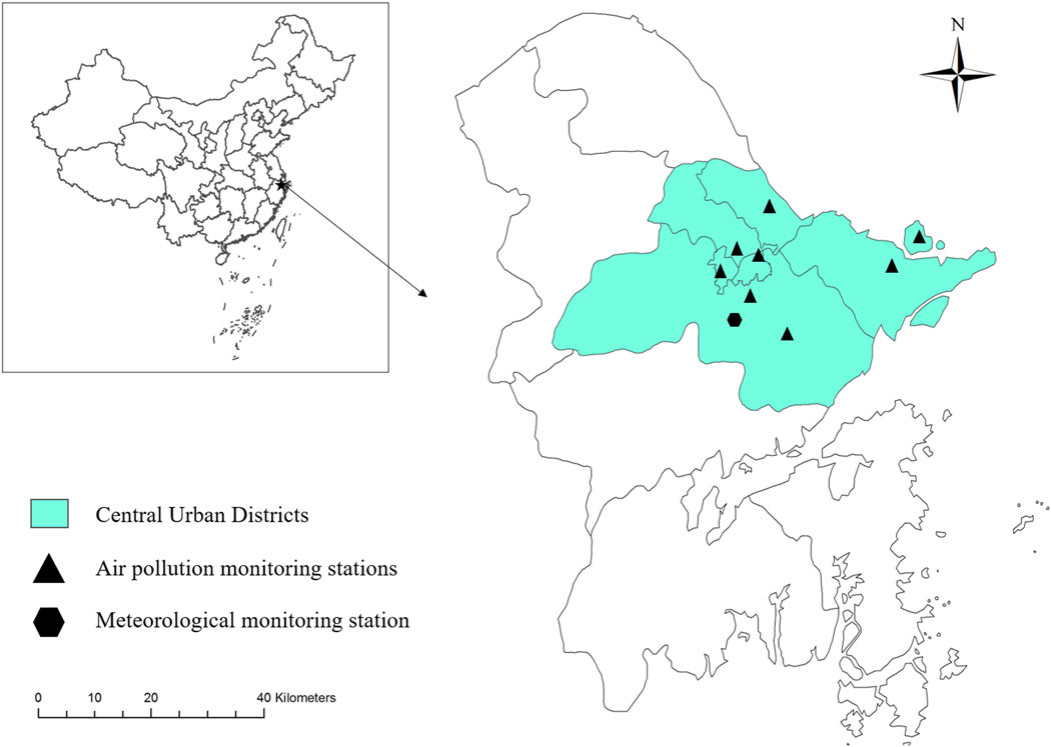
The spatial distribution of environmental monitoring stations in Ningbo city.

**Fig. 2.**
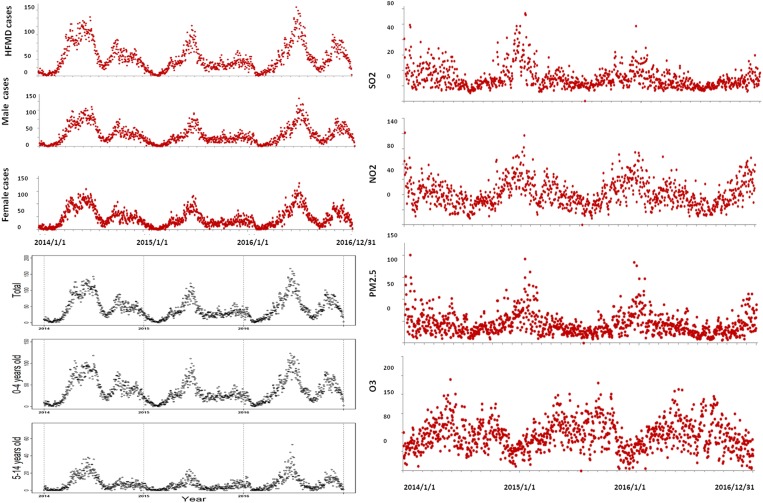
Raw plots showing the temporal trends of HFMD cases in Ningbo city, 2014–2016.

During the period from 2014 to 2016, a total of 48 209 HFMD cases occurred in Ningbo city, 28 606 (59.34%) of which were male and 19 603 (40.66%) female; about 82.88% of the cases were children less than 5 years old. Figure 1 shows the spatial distribution of environmental monitoring stations while Figure 2 illustrates daily trends in the distribution of HFMD cases and levels of air pollutants. We observed two peaks of HFMD cases in Ningbo city in April/July and September/November, and the seasonal distribution of all population groups was similar. The summary statistics of HFMD, meteorological factors and air pollution can be found in Table 1.

**Table 1.** Summary statistics of HFMD, meteorological factors and air pollution in Ningbo city, during 2014–2016

Groups	Mean	s.d.	Percentile		
			P1	P50	P99
All age	44.0	32.4	2.0	35.0	134.0
Sex					
Male	26.1	19.5	0.0	21.0	80.0
Female	17.9	13.6	0.0	14.5	55.0
Age					
0–4 years	36.5	25.9	1.0	30.0	106.0
5–14 years	7.5	7.4	0.0	5.0	33.1
Environment factors					
PM_2.5_ (μg/m^3^)	43.2	26.4	9.7	37.0	133.4
SO_2_ (μg/m^3^)	41.1	17.5	12.4	38.1	89.9
NO_2_ (μg/m^3^)	15.2	7.8	6.6	13.1	44.6
O_3_-8 h (μg/m^3^)	94.0	40.1	13.1	90.0	197.7
Average temperature (°C)	17.9	8.2	2.0	19.1	31.3
Relative humidity (%)	76.8	11.5	46.0	78.0	96.0
Sunshine duration (h)	4.1	3.9	0.0	3.4	11.6

**Fig. 3.**
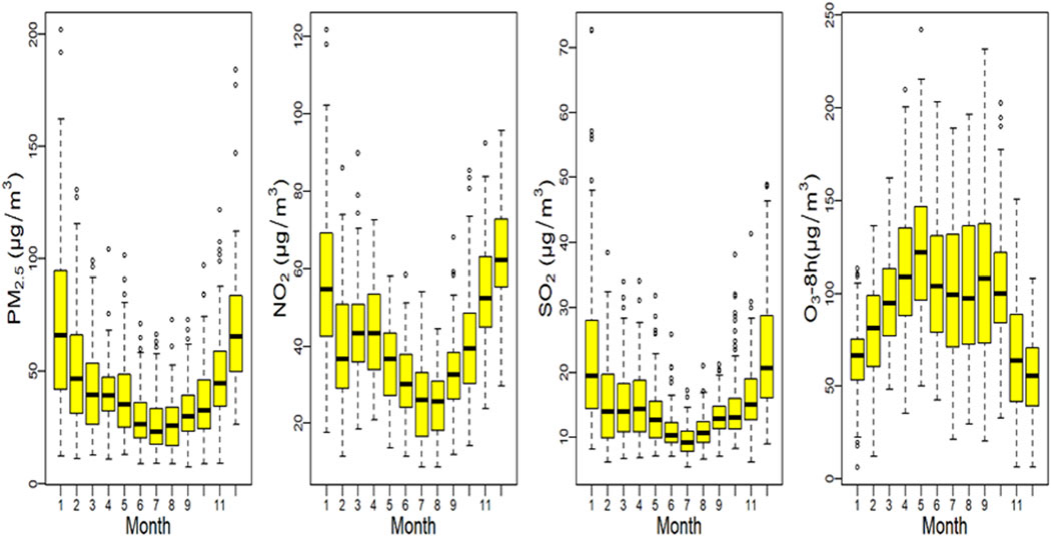
Boxplots of monthly air pollution in Ningbo city, during 2014–2016.

**Fig. 4.**
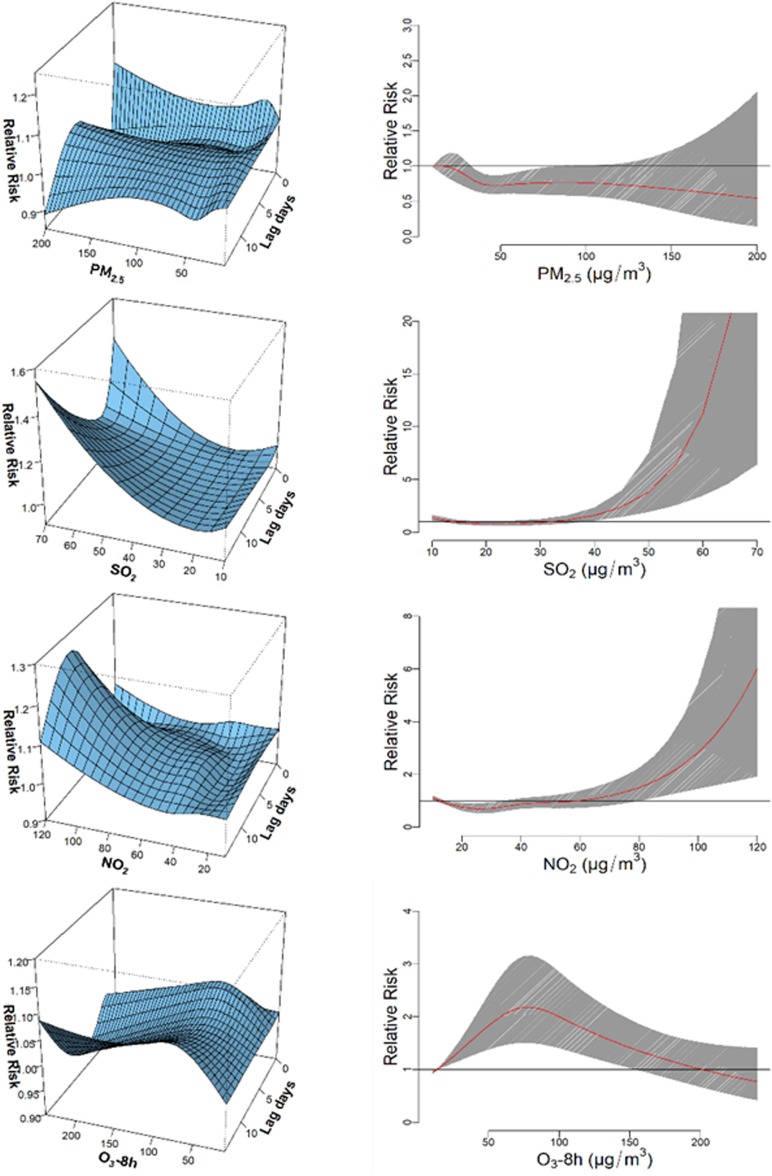
Association between air pollution and HFMD cases. Left panels: 3-D graphs of the exposure-lag-response risk surface. Right panels: overall cumulative exposure–response associations.

Figure 3 shows boxplots of monthly ambient air pollutant levels. The concentrations of PM_2.5_, SO_2_ and NO_2_ peaked in winter, with low values in summer, while the distribution of O_3_-8 h showed the opposite pattern. Table 2 shows the Spearman correlation coefficients for comparisons between different environment factors. Coefficients for relationships among PM_2.5_, SO_2_ and NO_2_ were high, while those among PM_2.5_, SO_2_ and O_3_-8 h were not statistically significant. Figure 4 shows the association between air pollution and HFMD cases. In general, we found positive associations between NO_2_, SO_2_, O_3_-8 h and the number of daily HFMD cases, but none for PM_2.5_. The exposure–response associations of NO_2_ and SO_2_ with HFMD cases were approximately linear, but the effect of O_3_-8 h decreased about 80 µg/m^3^. The overall estimated relative risk (RR) values for moderate levels of PM_2.5_, SO_2_, NO_2_ and O_3_-8 h with a lag of 14 days were 0.77 (0.64–0.92), 1.06 (0.87–1.29), 0.85 (0.68–1.07) and 2.12 (1.47–3.05), respectively. The overall RR estimates for high levels of PM_2.5_, SO_2_, NO_2_ and O_3_-8 h with a lag of 14 days were 0.71 (0.46–1.09), 2.32 (1.42–3.79), 2.01 (1.22–3.31) and 1.02 (0.69–1.53), respectively (Table 3).

**Table 2.** The spearman correlations between different environment factors

Factors	PM_2.5_ (μg/m^3^)	SO_2_ (μg/m^3^)	NO_2_ (μg/m^3^)	O_3_-8 h (μg/m^3^)	Temperature (°C)	Humidity (%)	Sunshine (h)
PM_2.5_ (μg/m^3^)	**1.000**						
SO_2_ (μg/m^3^)	**0.773**	**1.000**					
NO_2_ (μg/m^3^)	**0.728**	**0.720**	**1.000**				
O_3_-8 h (μg/m^3^)	0.026	0.046	**−0.242**	**1.000**			
Temperature (°C)	**−0.464**	**−0.483**	**−0.579**	**0.376**	**1.000**		
Humidity (%)	**−0.230**	**−0.437**	−0.032	**−0.394**	**0.183**	**1.000**	
Sunshine (h)	0.049	**0.165**	**−0.149**	**0.491**	**0.166**	**−0.687**	**1.000**

*Note*. Statistically significant (*P* < 0.05) were labelled in bold font.

**Table 3.** The overall estimated RR values for air pollutants at different levels with a lag of 14 days

	Moderate level	High level
PM2.5	0.77 (0.64–0.92)	0.71 (0.46–1.09)
SO_2_,	1.06 (0.87–1.29)	2.32 (1.42–3.79)
NO_2_	0.85 (0.68–1.07)	2.01 (1.22–3.31)
O_3_-8 h	2.12 (1.47–3.05)	1.02 (0.69–1.53)

Tables 4 and 5 show the effects of moderate and high levels of air pollution on HFMD cases in various population groups. We found that the effects of SO_2_ and NO_2_ were stronger in children less than 5 years old, with overall RR values for high levels thereof 2.39 (1.44–3.96) and 2.02 (1.21–3.39), respectively. On the other hand, the effects of O_3_-8 h were stronger in children aged 5–14 years, and the overall RR for high levels of O_3_-8 h was 2.31 (1.09–4.89).

**Table 4.** Cumulative relative risk (RR) and 95% confidence intervals (95% CI) for medium levels of air pollution on HFMD over a lag of 14 days

Groups	PM_2.5_	SO_2_	NO_2_	O_3_-8 h
All age	**0.77 (0.64–0.92)**	1.06 (0.87–1.29)	0.85 (0.68–1.07)	**2.12 (1.47–3.05)**
Sex				
Male	**0.78 (0.62–0.97)**	1.05 (0.83–1.33)	0.84 (0.65–1.10)	**2.01 (1.30–3.11)**
Female	**0.76 (0.60–0.97)**	1.08 (0.83–1.40)	0.91 (0.68–1.23)	**2.31 (1.44–3.71)**
Age, years				
0–4	**0.74 (0.61–0.90)**	1.02 (0.83–1.26)	0.85 (0.67–1.08)	**1.82 (1.24–2.67)**
5–14	0.89 (0.62–1.27)	1.23 (0.83–1.80)	0.93 (0.60–1.43)	**4.20 (2.12–8.33)**

*Note*. Statistically significant (*P* < 0.05) were labelled in bold font. The reference values were the 1st percentiles of each air pollutant. The effects of medium levels of air pollution were estimated by calculating the risk of HFMD at the 50th percentiles of each air pollutants relative to the reference.

**Table 5.** Cumulative relative risk (RR) and 95% confidence intervals (95% CI) for high levels of air pollution on HFMD over a lag of 14 days

Groups	PM_2.5_	SO_2_	NO_2_	O_3_-8 h
All age	0.71 (0.46–1.09)	**2.32 (1.42–3.79)**	**2.01 (1.22–3.31)**	1.02 (0.69–1.53)
Sex				
Male	0.79 (0.47–1.31)	**2.38 (1.33–4.25)**	**2.23 (1.24–4.01)**	1.06 (0.66–1.70)
Female	0.62 (0.36–1.10)	**2.20 (1.15–4.18)**	1.82 (0.83–1.40)	1.02 (0.60–1.71)
Age, years				
0–4	0.73 (0.47–1.15)	**2.39 (1.44–3.96)**	**2.02 (1.21–3.39)**	0.88 (0.58–1.33)
5–14	0.59 (0.26–1.32)	1.86 (0.71–4.89)	2.21 (0.85–5.76)	**2.31 (1.09–4.89)**

*Note*. Statistically significant (*P* < 0.05) were labelled in bold font. The reference values were the 1st percentiles of each air pollutant. The effects of high levels of air pollution were estimated by calculating the risk of HFMD at the 99th percentiles of each air pollutants relative to the reference.

## Discussion

HFMD affects children, in particular children less than 5 years old, resulting in serious complications including pneumonia and even death. As currently no effective vaccine is available, seeking risk factors and improving prevention strategies are crucial for children health. Although previous studies have indicated that temperature and some air pollutants including PM_10_ are associated with risk of HFMD [18, 29, 30], few focus on analysis of the effects of PM_2.5_ accompanied by the other air pollutants such as SO_2_, NO_2_ and O_3_. The present study included 48 209 HFMD cases that occurred from 2014 to 2016 in Ningbo city. The DLNM study indicated associations between the daily incidence of HFMD and air pollutants, including SO_2_, NO_2_, O_3_ and PM_2.5_. The data also suggested that high SO_2_ levels had an elevated RR of 2.38 (95% CI 1.33–4.25) among male children, compared with 2.20 (95% CI 1.15–4.18) among female children. Specifically, high levels of SO_2_ and NO_2_ had RRs of 2.39 (95% CI 1.44–3.96) and 2.02 (95% CI 1.21–3.39), respectively, among 0–4-year-old children, while O_3_ had an RR of 2.31 (95% CI 1.09–4.89) among 5–14-year-old children. Our findings suggest significant associations of high SO_2_ and NO_2_ levels and moderate O_3_ levels with HFMD epidemics, and indicate that air pollution has lagged effects on HFMD epidemics.

We found that the overall RR for moderate levels of SO_2_ with a lag of 14 days was 1.06 (95% CI 0.87–1.29) while that for high levels was 2.32 (95% CI 1.42–3.79), which is consistent with previous research [31]. SO_2_ is a toxic gas that is released naturally from volcanic activity and produced from the burning of fossil fuels, heavy metal extraction and chemical industries [32]. SO_2_ is a major air pollutant and has been reported to impact children's health. A study by Kathuria *et al*. indicated that the prevalence and severity of eczema in children in the United States is associated with higher mean annual SO_2_ levels [33]. Le *et al*. demonstrated that in Ho Chi Minh City, Vietnam, hospital admissions of young children for acute lower respiratory infection were generally positively correlated with ambient levels of SO_2_ during the dry season (November–April), but not the rainy season (May–October) [34]. In China, Song *et al*. explored the acute effects of SO_2_ on children's outpatient visits for respiratory diseases and found that a 10 µg/m^3^ increase in the 2-day average concentration (lag01) of SO_2_ corresponded to an increase of 0.33% (95% CI 0.10–0.56%) in daily hospital visits [35].

In addition to SO_2_ pollution, we also explored the effect of NO_2_ on the HFMD incidence. Compared with moderate levels of air pollution, the high NO_2_ level had an RR of 2.01 (95% CI 1.22–3.31). We also found that high levels of NO_2_ had an RR of 2.02 (95% CI 1.21–3.39) among 0–4-year-old children. These results indicate that NO_2_ is associated with an increased risk of HFMD, particularly in children under 5 years old. NO_2_ is a key component of air pollution, is predominantly caused by traffic exhaust. A number of studies have reported that NO_2_ is associated with childhood respiratory diseases. Lai *et al*. found that NO_2_ exposure was associated with an increased number of thunderstorm asthma related visits to health services. It is hypothesised that NO_2_ acutely exacerbates asthma, resulting in respiratory system sensitivity [36]. A study by Finke *et al*. indicated that NO_2_ exposure is related to lower pulmonary function among school children [37]. Kravitz-Wirtz *et al*. showed that early life NO_2_ exposure is associated with subsequent cases of childhood asthma [38]. However, some studies found the opposite results regarding this pollutant. A study published in 2009 found no significant cross-sectional association between NO_2_ concentrations and respiratory and allergic disorders in adults [39]. Lan *et al*. investigated the effects of NO_2_ on children's respiratory health and found that children's lung function indicators, such as forced vital capacity, were inversely correlated with annual NO_2_ levels (−0.0023 l/μg per m^3^; −0.0044 to −0.0002; *P* = 0.033) [40]. Although the results of our study demonstrated that NO_2_ is positively associated with childhood HFMD incidence in Ningbo City, there have been few studies regarding the association between NO_2_ and HFMD in children to date. Considering that Ningbo City is an industrialised city with a large number of factories and vehicles, and the annual NO_2_ concentration is 41.06, which is above the national environmental air quality standard limit of 40 µg/m^3^, large-scale actions to reduce emissions of NO_2_ and guidelines to protect 0–5 year-old children from its effects are urgently needed.

In addition to NO_2_ and SO_2_, we included O_3_ in the DLNM so that we could explore its potential effects on HFMD incidence. O_3_ is a potent airway irritant as well as a factor affecting respiratory morbidity, especially in young people. According to a study conducted in three cities in the U.S., O_3_ is significantly related to pediatric respiratory morbidity (OR 1.08, 95% CI 1.06–1.11) [41]. High O_3_ levels were reported to have a protective effect against HFMD in Guilin City, China [17]. In contrast, some previous studies reported no association between O_3_ exposure and respiratory diseases in children. For example, Li *et al*. reported no association between O_3_ exposure and upper respiratory tract infection in hospital outpatients aged 0–14 years in Hefei Province, China [42]. Our study supports previous findings that O_3_ was associated with an increased risk of HFMD in children. In our results, The RR of O_3_ was 2.12 (95% CI 1.47–3.05) at moderate levels of air pollution; specifically, high levels of O_3_ had an RR of 2.31 (95% CI 1.09–4.89) among 5–14-year-old children. Possible explanations for this discrepancy include that the O_3_ concentration in Ningbo City may be different from that in other regions, or that other air pollutants may interact with O_3_. Liang L *et al*. analysed the association between dairy SO_2_, NO_2_, O_3_, CO, PM_10_ and PM_2.5_ and hospitalisations for acute exacerbation of COPD in Beijing from 2013 to 2017 and the results indicated that exposure–response association of NO_2_ with COPD cases was linear, while the SO_2_, CO, PM_10_ and PM_2.5_ were non-linear, no significance was found in O_3_ with COPD incidence [43]. Liu C *et al*. investigated the air pollution and daily mortality in 652 cities, the results demonstrated that exposure–response association of PM_2.5_ with mortality was non-linear [26]. However, till now, the exposure–response of associations of air pollutants and some certain diseases are not well consistent. The possible reasons may be due to the different study regions, different air pollutants' concentrations or other confounder factors such as differential culture background. Therefore, in the future, the studies should be conducted at multiple-cities levels.

PM_2.5_, another potent airway irritant, is reported to be closely related to HFMD incidence in Guilin City, China. Low PM_2.5_ levels decrease the risk of HFMD, whereas high levels increase the incidence of HFMD [17]. Our previous studies on the effects of PM_10_ on HFMD in Ningbo City found no significant correlation between PM_10_ and HFMD incidence, except in females [18, 44]. Yu *et al*. indicated that low PM_2.5_ levels had a protective effect on HFMD incidence, as the corresponding RR value was 0.85 (95% CI 0.74–0.98), which is similar to our results. In this study, we found that PM_2.5_ was a protective factor against HFMD in children, with an RR of 0.77 (95% CI 0.64–0.92) at moderate levels of air pollution. The discrepancy among these studies may be explained as follows. The PM_2.5_ level varies among different regions; for instance, the mean PM_2.5_ concentration in Guilin City is 52.8 µg/m^3^, whereas in Ningbo City it is 43.15 µg/m^3^. The former is located in western China, whereas the latter is adjacent to the sea, where the east sea wind may decrease the concentration of PM_2.5_. Another reason may be that air pollutants form a complex mixture of compounds, thus confounding each other's effects. Third, low levels of PM_2.5_ exposure may stimulate the immune system, while high levels may compromise immune function, as environmental genotoxicants have been shown to exert different effects (i.e. harmful or beneficial) depending on molecular and ecological factors [45–47]. This study shows that the impacts of these air pollutants on HFMD are complex and require further exploration, particularly of the underlying mechanisms. Meanwhile, policy makers should pay more attention to children aged less than 5 years, as they are more sensitive to air pollutants than older children.

We should mention the limitations of this study. First, most HFMD cases were diagnosed clinically rather than by laboratory tests, which may have biased the data. Second, only 3 years of data were analysed. Third and the major limitation of the time-series study is that we only use the concentration of air pollution at the city level, not at the individual level, which may cause ecological bias. In addition, individuals have different activity levels, which might lead to significant biases in exposure assessments. Despite these limitations, this is the first investigation of the effect of NO_2_ on HFMD incidence. Our results will further our understanding of the effects of air pollutants on vulnerable populations.

## Conclusions

Our findings suggest significant associations of high SO_2_ and NO_2_ levels and moderate O_3_ levels with HFMD epidemics, and indicate that air pollution has a lagged effect on HFMD epidemics. Furthermore, compared with females and 5–14-year-old children, males and 0–4-year-old children are at higher risk of contracting HFMD. Our study provides useful information for targeted prevention and control measures based on environmental evidence.

## Data

Raw data of HFMD incidence and daily air pollution levels were requested and obtained from Ningbo Municipal Center for Disease Control and Prevention and the Environment Monitoring Center of Ningbo, respectively. The raw meteorological data were obtained from the Ningbo Meteorological Bureau. Raw data will not be shared because the authors are not authorised for distribution of data.
